# Notes and Notices

**Published:** 1894-10

**Authors:** 


### NOTES AND NOTICES.

ROSEDALE, Lester Wallack's successful play, will be presented at the Chestnut Street Opera House, Philadelphia, during the week commencing Monday, September 24th, with Joseph S. Haworth as the stellar attraction, and a cast including the following well-known favorites: M. A. Kennedy, Chas. B. Hanford, Chas. Abbott, Isabelle Evesson, Maud Haslam, and Mrs. E. A. Eberle.

None of the appointments of a first-class production will be lacking. The famous ball-room and gypsy dell scenes will be seen to unusually good advantage. "Rosedale" comes direct from the Star Theatre, New York, where it is now enjoying one of the most successful engagements at that theatre of recent years.

Since the above was set in type the editor has witnessed this play and can cordially indorse it as being interesting, natural in the situations, and free from everything that would offend any one's sense of propriety.

The scenery and general appointments are all that are promised and the acting superb. The *Elliot Gray* of Mr. Joseph S. Haworth is a fine rendering of the brave and manly soldier full of resources in the most desperate situations, and Mr. Abbott as the villain Miles McKenna was especially interesting.

THE PEOPLE'S HEALTH JOURNAL, of Chicago, offers *one hundred dollars* for the best original story contributed to its columns before January 1st, 1895. The story must contain not less than 2,500 words, nor more than 3,500. It must illustrate incidentally, hygiene, diet, dress, Homœopathy, and medicine in general. Among the characters there must be a doctor, a nurse, and a patient. Those intending to compete for this liberal prize should write for full particulars.

A Huge Pile of Confederate Money.—Eighty million dollars in bills were shipped to Atlanta yesterday, the mammoth packages of money filling five large dry goods boxes and making in all more than a dray load. None of the bills are current, however, as they represent "nothing in God's earth now and naught in the waters below it." They were Confederate bills of the rarest type.

The huge pile of genuine Confederate money was shipped here from Richmond, Va., the former capital of the Confederacy, and is now the property of Mr. Chas. D. Barker, No. 90 S. Forsyth Street, this city. The money is of every denomination issued by the departed nation, and in the big collection are bills of the rarest type. There are bills issued during every year of the war. Thousands of them are very valuable as relics, but the great number of them Mr. Baker has on hand will make them so common as to bring but little on the market.

This eighty millions of dollars of Confederate money has been all along supposed to have been destroyed. This is undoubtedly the largest lot of Confederate money in the world.—Atlanta, Ga., *Constitution*, June 4th.

Dr. Walter H. Phillips, formerly house physician to the Children's Homœopathic Hospital, Philadelphia, Pa., has removed to Thomasville, Ga., where he will devote his attention to throat and lung troubles. Physi ians who wish to send patients afflicted with lung troubles to the South to avoid Northern winters should commend them to the care of Dr. Phillips at Thomasville.

Meeting of Medical Publishers.—The first annual meeting of the American Medical Publishers' Association occurred at Hot Springs, Va., on August 13th and 14th, and proved a most profitable and happy event, many of the members being accompanied by their ladies, who enjoyed the pretty scenery and lent a graceful charm to the occasion. An overland trip to Warm Springs comprised a pleasant feature of the first day. After the transaction of the usual routine business, the President, Dr. Landon B. Edwards, read an able paper on "Advertising and Advertising Agencies," which was well received and ordered printed. The reports of the Secretary and Treasurer were examined, and the finances of the Association found to be in good condition. Thirteen applications for membership were presented and acted upon favorably. The Secretary was instructed to investigate the laws of different States governing charters of incorporated bodies, and report at the next meeting. Dr. J. W. Clausen, of the *Times and Register*, Philadelphia, was elected to fill a vacancy in the Executive Committee. Upon motion, it was decided that all annual meetings hereafter should be held just prior to the sessions of the American Medical Association, the next meeting being set for Monday, June 5th, 1895, at 9 A. M., in the Utah House, Baltimore, Md.

Charles Wood Fassett, *Secretary.*

